# Structural and functional studies of SAV0551 from *Staphylococcus aureus* as a chaperone and glyoxalase III

**DOI:** 10.1042/BSR20171106

**Published:** 2017-11-17

**Authors:** Hyo Jung Kim, Ki-Young Lee, Ae-Ran Kwon, Bong-Jin Lee

**Affiliations:** 1 Research Institute of Pharmaceutical Sciences, College of Pharmacy, Seoul National University, Gwanak-gu, Seoul 151-742, Korea; 2 Department of Herbal Skin Care, College of Herbal Bio-industry, Deagu Haany University, Gyeongsan 712-715, Korea

**Keywords:** crystallography, DJ-1 superfamily, glyoxalase, Hsp31, molecular chaperones, staphylococcus aureus

## Abstract

The DJ-1/ThiJ/*Pf*pI superfamily of proteins is highly conserved across all biological kingdoms showing divergent multifunctions, such as chaperone, catalase, protease, and kinase. The common theme of these functions is responding to and managing various cellular stresses. DJ-1/ThiJ/*Pf*pI superfamily members are classified into three subfamilies according to their quaternary structure (DJ-1-, YhbO-, and Hsp-types). The Hsp-type subfamily includes Hsp31, a chaperone and glyoxalase III. SAV0551, an Hsp-type subfamily member from *Staphylococcus aureus*, is a hypothetical protein that is predicted as Hsp31. Thus, to reveal the function and reaction mechanism of SAV0551, the crystal structure of SAV0551 was determined. The overall folds in SAV0551 are similar to other members of the Hsp-type subfamily. We have shown that SAV0551 functions as a chaperone and that the surface structure is crucial for holding unfolded substrates. As many DJ-1/ThiJ/*Pf*pI superfamily proteins have been characterized as glyoxalase III, our study also demonstrates SAV0551 as a glyoxalase III that is independent of any cofactors. The reaction mechanism was evaluated via a glyoxylate-bound structure that mimics the hemithioacetal reaction intermediate. We have confirmed that the components required for reaction are present in the structure, including a catalytic triad for a catalytic action, His^78^ as a base, and a water molecule for hydrolysis. Our functional studies based on the crystal structures of native and glyoxylate-bound SAV0551 will provide a better understanding of the reaction mechanism of a chaperone and glyoxalase III.

## Introduction

The DJ-1/ThiJ/*Pf*pI superfamily (DJ-1 superfamily hereinafter) is a group of proteins that are universal to all the kingdoms of life [1]. The members of this superfamily share structural and functional similarity to human DJ-1, which is known to be associated with Parkinson disease [2,3]. The crystal structures of several members of the DJ-1 superfamily have been solved. Most of them are oligomers, with each monomer consisting of center-aligned β-strands surrounded by α-helices, which is called an α/β sandwich fold [4]. A completely conserved cysteine is located at a sharp turn between a β-strand and an α-helix. The sharp turn motif is referred to as the ‘nucleophilic elbow’ and is known to allow easy access to the cysteine by substrates [5]. DJ-1 superfamily proteins are divided into the following distinct subfamilies based on their quaternary structure: (i) DJ-1-type, (ii) YhbO-type, and (iii) Hsp-type. Each of the DJ-1 subfamilies also differs in the architecture of the conserved cysteine. DJ-1-type subfamily proteins (DJ-1 and YajL) form dimeric interfaces consisting of α-helices, β-strands, and loops. The conserved cysteine does not form a catalytic triad in human DJ-1 and bacterial YajL [6,7]. Many functions of DJ-1 have been revealed including chaperone, neurone protection, and glyoxalase III [8–10]. The YhbO-type subfamily includes the representative YhbO from *Escherichia coli*, SAV1875 from *Staphylococcus aureus, Pf*pI from *Pyrococcus horikoshii*, and Ton1285 from *Thermococcus onnurineus*. YhbO-type subfamily members form dimers with three α-helices. The catalytic triad in this subfamily consists of a cysteine, histidine, and an acidic residue from the adjacent subunit. Ton1285 and *Pf*pI from the YhbO-type subfamily are known to cleave peptide bonds [4,11]. Hsp-type subfamily members have a unique dimerization surface consisting of N-terminal β-strands and loops [12]. As heat shock proteins (HSPs) are considered molecular chaperones, most members of the Hsp-type subfamily function as chaperones, specifically holding chaperones [13]. Holding chaperones capture unfolded proteins with their surface structures for proper folding. The discriminating surface structures, canyons, and bowls of Hsp31 from *E. coli* and *Vibrio cholerae* distinguish them from other subfamily members [12,14]. Hsp-type DJ-1 subfamily proteins have a catalytic triad consisting of a cysteine, histidine, and an acidic residue from the cap domain of the same subunit. With the catalytic cysteine in the catalytic triad, most proteins in the Hsp-type subfamily function as glyoxalase III [15–18].

The distinct subclass classification correlates with structural characteristics, but known functions of DJ-1 superfamily proteins cannot be classified into subclasses. From our previous study, we determined the surface structure of the YhbO-type protein SAV1875 differs from the YhbO-type subfamily proteins but is similar to Hsp-type subfamily proteins. SAV1875 shows chaperone activity similar to other Hsp-type subfamily proteins [19]. However, the Hsp-type protein Glx3 from *Candida albicans* and YDR533C from *Saccharomyces cerevisiae* do not show chaperone activity. The differences in the chaperone activity within the Hsp-type subfamily are shown to be based on the importance of their surface structure, as these two proteins lack the 45 amino acid N-terminal region [16]. The glyoxalase III function was first revealed in Hsp-type subfamily proteins, though YajL and DJ-1 from the DJ-1-type and YhbO from the YhbO-type subfamily also show the same function [9,20]. Despite the structural and functional differences in the classes, many DJ-1 superfamily proteins perform chaperone and glyoxalase III functions. Under stress conditions, chaperone proteins protect cells by stabilizing unfolded proteins, giving the cell time to repair or resynthesize damaged proteins, which is important for protecting cells from severe stress conditions [21]. Glyoxalase III directly converts the toxic methylglyoxal substrate to non-toxic D-lactate without glutathione. Methlyglyoxal is a toxic endogenous electrophile component that damages proteins, nucleic acids, and lipids [22]. Therefore, studying the DJ-1 superfamily of proteins is important for understanding cellular protection mechanisms from various stress conditions.

In the present study, the structural and functional identification of hypothetical SAV0551 from *S. aureus* Mu50 was conducted. *S. aureus* Mu50 (ATCC 700699) is a vancomycin-intermediate *S. aureus* that displays robust virulence properties. It causes severe infectious diseases ranging from mild infections, such as skin infections and food poisoning, to life-threatening infections, such as sepsis, endocarditis, and toxic shock syndrome [23]. However, because of the bacterial resistance, the prognosis for *S. aureus* infection is still poor despite early diagnosis and appropriate treatment [24]. Although there are various theories regarding how *S. aureus* acquires antibiotic resistance, very little is known. In this regard, understanding the bacterial defense mechanisms in *S. aureus* against antibiotics is crucial. Furthermore, the DJ-1 superfamily of proteins is predicted to have stress-response functions under multiple stress conditions [25]. Therefore, we designed structural and functional experiments with SAV0551, a DJ-1 superfamily protein from *S. aureus* Mu50, as an extension of our previous work on SAV1875. We elucidated the structure and function of SAV0551 as a chaperone and glyoxalase III. The catalytic triad mutants (C190A, H191A, and D221A) and absolutely conserved glutamate mutant (E81A) were designed to identify structure-related functional differences. The mutants lost glyoxalase III activity but maintained chaperone activity. We show a co-crystal structure of SAV0551 with the known glyoxalase III inhibitor glyoxylate, and it shows the intermediate structure in the glyoxalase III reaction. Our structural and functional research on previously unknown SAV0551 presents new residues that are involved in glyoxalase III reaction.

## Experimental

### Cloning, protein expression, and purification

The predicted ORF of SAV0551 was amplified from *S. aureus* Mu50 genomic DNA using standard PCR methods. The forward and reverse oligonucleotide primers were designed using the published genome sequence, 5′- GACTGCATATGTCACAAGATGTAAATGAATTAAG and 5′- GTCACTCGAGT TTATTTTGTATTGCATTTAACAT, respectively, where the bases underlined represent the NdeI and XhoI restriction enzyme cleavage sites. The amplified DNA was inserted into the NdeI/XhoI-digested expression vector pET-21a(+) (Novagen). The resulting construct contains eight non-native residues at the C-terminus (LEHHHHHH) that facilitate protein purification. The accuracy of the cloning was confirmed by DNA sequencing. The resulting expression plasmid was then transformed into *E. coli* BL21(DE3) cells (Novagen).

To prepare mutants, the EZchange Site-directed Mutagenesis kit (Enzynomics) was used to generate point mutations in the SAV0551 recombinant pET-21a(+) plasmid. The point mutations resulted in separate multiple recombinant plasmids, specifically E81A, C190A, H191A, and D221A. The sequences of the reconstructed mutants were confirmed by DNA sequencing (results not shown).

The wild-type and SAV0551 mutants’ (E81A, C190A, H191A, and D221A) cells were grown at 37°C until the OD600 reached 0.6 and expression was induced by the addition of IPTG to a final concentration of 0.5 mM. After an additional 4 h of growth at 37°C, cells were harvested by centrifugation and resuspended in 50 mM Tris, pH 7.5, 0.5 M NaCl, and 20 mM imidazole buffer. Cells were lysed by sonication at 4°C and the supernatant was loaded on to Ni^2+^-NTA (Ni^2+^-nitrilotriacetate) affinity column (Qiagen; 3 ml of resin per liter of cell culture) previously equilibrated with the same buffer. The column was washed extensively with wash buffer (50 mM Tris, pH 7.5, 0.5 M NaCl, and 50 mM imidazole); then the bound protein was eluted with elution buffer (50 mM Tris, pH 7.5, 0.5 M NaCl, and 500 mM imidazole) until there was no detectable absorbance at 280 nm in the elutant. Fractions containing protein were concentrated to ~2 ml and applied to a Superdex 75 (10/300 GL) column (GE Healthcare Life Sciences) that had been equilibrated with the final buffer (50 mM Tris, pH 7.5 and 0.15 M NaCl). The purities of SAV0551 was judged to be over 95% by SDS/PAGE. The protein solution was concentrated using 10000 Da molecular-mass cut-off spin columns (Millipore). The protein concentration was estimated by measuring the absorbance at 280 nm, employing the calculated extinction coefficient of 35535 M^−1^.cm^−1^ (Swiss-Prot; http://www.expasy.org).

### Crystallization, data collection, structure determination, and refinement

Crystallization was performed at 293 K by the hanging-drop vapor diffusion method using 24-well VDX plates (Hampton Research). Initial crystallization conditions were established using screening kits from Hampton Research (Crystal Screens I and II, Index, PEG/Ion, and MembFac) and from Emerald BioSystems (Wizard I, II, III, and IV). For the optimal growth of the SAV0551 crystals, each hanging drop was prepared on a siliconized cover slip by mixing 1 μl of protein solution (15 mg/ml) and 1 μl of precipitant solution (23% (w/v) PEG3350, 100 mM BisTris, pH 6.0), and this drop was equilibrated against a 1-ml reservoir of precipitant solution. These conditions yielded needle-shaped crystals for each protein that grew to dimensions of 1.0 × 0.3 × 0.2 mm in two days. The co-crystal with glyoxylate were prepared by addition of 50 mM glyoxylate, 0.2–1 μl of protein solution and 1 μl of precipitant solution (30% (w/v) PEG3350, 100 mM BisTris, pH 6.5). All crystals belonged to space group P2_1_2_1_2_1_ and contained eight molecules per asymmetric unit.

For crystal freezing, the crystals were transferred to a cryoprotectant solution with 30% (v/v) ethylene glycol in the crystallization condition for several minutes before being flash-frozen in a stream of nitrogen gas at 100 K. Diffraction data were collected on beamline 5C at the Pohang Light Source, South Korea. The raw data were processed and scaled using the HKL2000 program suite [26]. Further data analysis was carried out using the CCP4 suite [27]. Data collection statistics are summarized in Table 1.

**Table 1:** Crystallographic data collection and refinement statistics

	SAV0551	SAV0551 with glyoxylate
**Data collection**		
Beamline	PAL-5C	PAL-5C
Wavelength (Å)	0.98	0.98
Resolution range^a^ (Å)	40.00-2.60	40.00-3.01
Space group	P2_1_2_1_2_1_	P2_1_2_1_2_1_
Unit cell parameters (Å)	a =96.28	a =97.20
	b =129.45	b =130.12
	c =187.85	c =187.95
Observations (total/unique)	472, 116/72, 192	590, 230/47, 559
Completeness (%)	99.3 (97.4)	99.8 (99.2)
R_sym_^b^ (%)	10.7 (43.7)	20.7 (78.3)
I/sigma	32.7 (5.7)	20.4 (4.1)
**Refinement**		
R_work_^c^ (%)	18.3	18.8
R_free_^c^ (%)	26.5	26.6
Protein atoms	17,906	17,836
Water molecules	292	7
Average *B* value (Å^2^)	40.4	44.0
r.m.s.d. bond (Å)	0.021	0.009
r.m.s.d. angle (°)	1.020	1.114
**Ramachandran analysis (%)**		
Favored region	94.3	92.9
Allowed region	5.4	6.7
Outliers	0.3	0.4

a Numbers in parentheses indicate the statistics for the last resolution shell.

b R_sym_ = Σ(|*I_hkl_*-<*I_hkl_*>|/Σ<*I_hkl_*>; where *I_hkl_*, single value of measured intensity of *hkl* reflection; <*I_hkl_*>, mean of all measured value intensity of *hkl* reflection.

c R_work_ = Σ(|*F_obs_-F_calc_*|/Σ*F_obs_*; where *F_obs_*, observed structure factor amplitude; *F_calc_*, structure factor calculated from model. R_free_ is computed in the same manner as R_work_, but from a test set containing 5% of data excluded from the refinement calculation.

To determine the structure of the wild-type SAV0551, molecular replacement was used with the program Molrep [28] within the CCP4 suite [27] using the homologous structure of Hsp31 from *V. cholerae* (PDB code: 4I4N) as a search model. The SAV0551 and Hsp31 from *V. cholerae* shows 49% sequence identity. To determine the structure of glyoxylate-bound SAV0551, molecular replacement was performed using the wild-type SAV0551 as the search model. Refinement of each crystal structure was done through iterative cycles of model building using COOT [29], followed by refinement of the models with Refmac5 and phenix.refine [30,31]. A 5% portion of the data were set aside prior to refinement for the R_free_ calculations for each dataset [32]. Solvent and glyoxylate molecules became apparent in the later stages of refinement and were added into the model. Further refinement was pursued until no further decrease in R_free_ was observed. Structural alignments were carried out using the program PyMOL (http://www.pymol.org) and UCSF Chimera (http://www.cgl.ucsf.edu/chimera) [33], which were then used for the construction and generation of all the figures. Protein interfaces, surfaces, and assemblies were calculated using the PISA server at the European Bioinformatics Institute (http://www.ebi.ac.uk/pdbe/prot_int/pistart.html) [34].

### Determination of chaperone activity

To monitor the chaperone activity of the wild-type and SAV0551 mutants, citrate synthase was employed as a substrate [35,36]. Initially, to identify chaperone activity, 75 μg of citrate synthase (Sigma–Aldrich) was mixed with a solution of 100 mM Tris, pH 8.0, 20 mM DTT, 6 M guanidine chloride (GnCl). The citrate synthase mixture (75 μg of citrate synthase, 100 mM Tris, pH 8.0, 6 M GnCl, 20 mM DTT) was incubated for 1 h at 25°C; consequently, the citrate synthase in this solution was denatured. After incubation, refolding of citrate synthase was achieved by 100-fold dilution with a solution of 100 mM Tris (pH 8.0) containing 5 μM wild-type and SAV0551 mutants. The diluted solution was mixed with acetyl-CoA, oxaloacetate, MnCl_2_ and 5,5′-dithiobis-(2-nitrobenzoic acid) (DTNB) to detect the activity of citrate synthase (100 mM Tris, pH 8.0, 1 mM DTNB, 0.2 mM MnCl_2_, 0.4 mM oxaloacetic acid, 0.3 mM acetyl-CoA). After mixing, only the active, refolded enzyme will break acetyl-CoA into the acetyl group and CoA. The CoA reacts with DTNB, which acts as a coloring agent, and this produces a yellow TNB-CoA-SH compound that is detectable at 412 nm using a Multi-Mode microplate reader (SpectraMax M5e) [37]. After a 25-min reaction period, the highest specific activity value obtained for TNB-CoA-SH was considered 100% and the lowest was 0%. The calculated specific activities of the wild-type and SAV0551 mutants were expressed as a percentage of this value.

### Determination of glutathione-independent glyoxalase (glyoxalase III) activity

The glyoxalase activity assay in the wild-type and SAV0551 mutants was performed using the methylglyoxal (Sigma–Aldrich, 40% solution) in a 1D NMR experiment with absorption spectroscopy. For NMR analysis of the SAV0551 enzymatic reactions, 60 mM methylglyoxal was mixed with 50 μM SAV0551 or the cysteine mutant (C190A). The reaction mixture was incubated at 37°C for 5 min in 20 mM HEPES buffer (pH 7.5) and included 50 mM KCl and 2 mM DTT. The NMR samples contained 10% D_2_O for deuterium locking. All the 1D NMR experiments were performed at 298 K on an 800 MHz Bruker Ascend NMR spectrometer equipped with a cryogenic probe. All spectra were acquired and processed using the Topspin 3.2 program. On-resonance presaturation pulses were used to suppress the water signal. 1H chemical shifts were internally referenced to 4,4-dimethyl-4-silapentane-1-sulphonic acid (DSS). The number of scans (NS) was 128 and the relaxation delay was 2 s, giving a total experimental time of ~9 min.

For absorption spectroscopy, the reactions were initiated by the addition of 6 mM methylglyoxal to the enzyme in the reaction buffer (20 mM HEPES, pH 7.5, 50 mM KCl, and 2 mM DTT). The methylglyoxal and protein (wild-type or SAV0551 mutants) reaction mixtures were incubated at 37°C. The final protein concentration was 10 μM. The remaining methylglyoxal at each time point was determined by reaction with 2,4-dinitrophenylhydrazine (DNPH) to generate the purple chromophore mehtylglyoxal-bis-2,4,-dinitrophenylhydrazone after alkali treatment [38]. The assay was performed by removing a 50 μl sample from the reaction at fixed time points after enzyme addition (0, 15, 30, 45, 60, 75, 90, 105, and 120 s) and rapidly mixing the sample with 0.9 ml of distilled water. To this solution, 0.33 ml of a freshly prepared stock of DNPH reagent (0.2% DNPH dissolved in 2 N HCl) was immediately added and incubated at 37°C for 15 min. This highly acidic solution stops the enzymatic reaction, whose rate was already greatly diminished by the initial 19-fold dilution into water. The purple color of the hydrazine is developed by the addition of 1.67 ml of 3.8 M NaOH and incubation for 10 min at room temperature followed by measurement of the absorbance at 550 nm in a SpectraMax M5e. All the measurements were made in triplicate.

### PDB codes

Protein co-ordinates and structure factors have been deposited in the RCSB PDB under codes 5XR2 for the native SAV0551 and 5XR3 for SAV0551 with glyoxylate.

## Results

### Crystal structure of SAV0551

**Figure 1:**
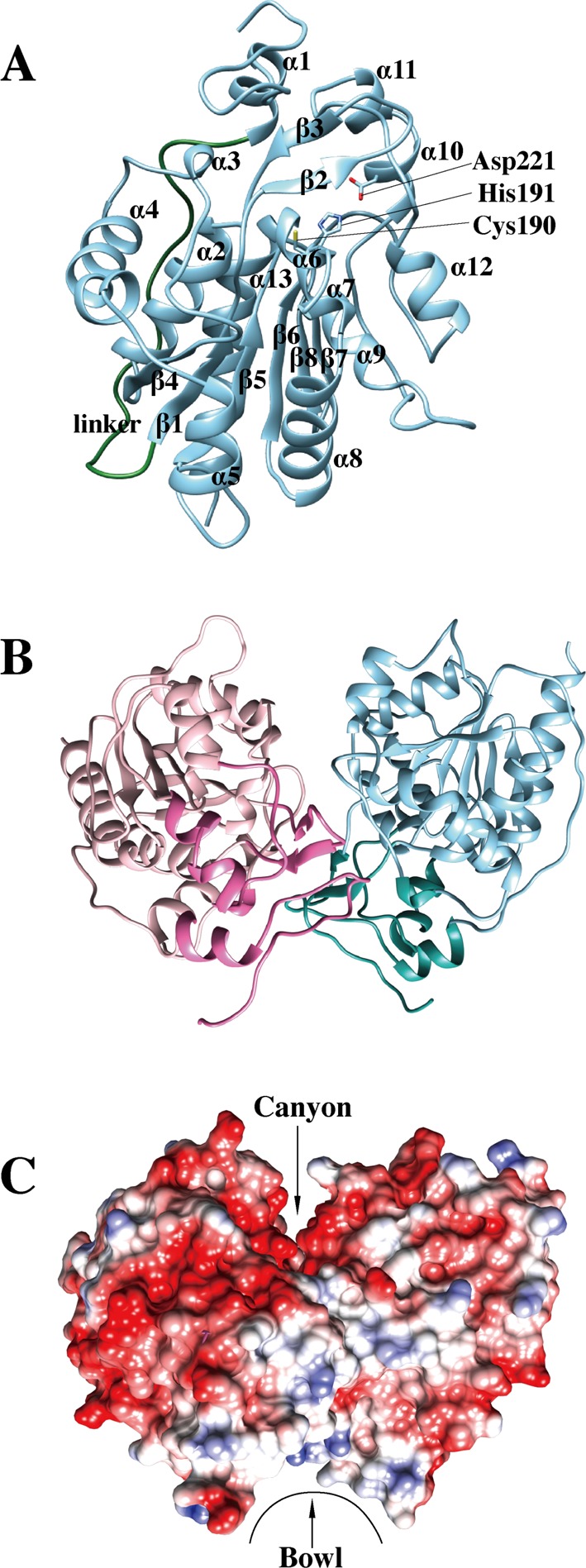
Crystal structure of SAV0551 (**A**) Ribbon representation of the SAV0551 monomer with two domains. SAV0551 shows a sandwich-folded main domain and a cap domain. Each domain is connected by a linker (green). SAV0551 has an α1-linker-β1-β2-β3-α2-β4-α3-α4-α5-β5-α6-α7-α8-β6-α9-α10-α11-α12-β7-β8-α13 topology. The Cys^190^ at a sharp turn between β6 and α9 forms a catalytic triad with His^191^ from the main domain and Asp^221^ from the cap domain. (**B**) The SAV0551 dimer is shown in ribbon representation from a side view. Chain A is colored in pink and chain B is colored in blue. The cap domain of each subunit is shown in a darker color. The β-strands and loop from the cap domain are mainly involved at the dimeric interface. (**C**) The potential surface charge of the SAV0551 crystal structure calculated by UCSF Chimera, where the surfaces are colored between –10 kcal/mol·e (red) and +10 kcal/mol·e (blue) (1 kcal =4.184 kJ). Most of the surface is negatively charged. The canyon and bowl are indicated.

The crystal structure of SAV0551 was determined at 2.6 Å resolution including the additional C-terminal histidine tag. The asymmetric unit contains eight molecules and each of them consists of two domains: a core domain whose similarity to DJ-1 superfamily proteins had been expected and a cap domain, which is also called the ‘P’ region. The core domain consists of an α/β sandwich fold with nine α-helices and six β-strands. Six β-strands, β4 (residues 95–100), β1 (residues 53–58), β5 (residues 151–156), β6 (residues 184–189), β8 (residues 265–268), and β7 (residues 259–262), are aligned in the center, wherein the latter β7 strand is antiparallel to the central β-strands. Nine helices surround the core of β-strands. α-helices α5 (residues 137–143), α6 (residues 160–163), α7 (residues 166–168), α8 (residues 170–181), α9 (residues 192–198), α12 (residues 239–245), α13 (residues 271–291), α2 (residues 79–91), and α4 (residues 118–132) are around the core in a clockwise direction. Additionally, four short α-helices (α1 (residues 26–32), α3 (residues 110–112), α10 (residues 218–222), and α11 (residues 224–227)) and two β-strands (β2 (residues 64–66) and β3 (residues 72–74)) form the cap domain. The core and cap domains are linked via a 22-residue long-linker (residues 33–53). Cys^190^ and His^191^ from the core domain and Asp^221^ from cap domain constitute a catalytic triad. The Cys^190^ S^γ^ interacts with the His^191^ N^δ1^, while the Asp^221^ carboxylate hydrogen bonds with His^191^ N^ε2^. This catalytic triad is well conserved in Hsp-type DJ-1 superfamily proteins (Figure 1A).

The crystal structure shows that SAV0551 is a compact homodimer. The loops (residues 1–25 and 67–71) and β-strands (β2 (residues 64–66) and β3 (residues 72–74)) from cap domain are responsible for dimer formation. Additionally, loops located in the core domain participate at the dimerization interface. The dimeric interface buries ~1110 Å^2^ per subunit. There are three charge–charge interaction pairs between Arg^63^ and Glu^18^, Lys^107^ and Glu^62^, and Lys^132^ and Asp^19^ on the dimeric interface of the cap domain. Hydrogen bonding interactions are observed at the dimeric interface of the core and cap domains and include Glu^18^, Asp^19^, Glu^61^, Glu^62^, Arg^63^, Ser^102^, Tyr^104^, and Lys^236^. The dimer crystal structure of SAV0551 is consistent with the results from size-exclusion chromatography, which showed a single band at ~64 kDa in solution (the molecular weight of a SAV0551 monomer is ~32 kDa) (Figure 1B).

The surface structure and electrostatic distribution of SAV0551 further display distinctive characteristics of the Hsp-type subfamily. The overall structure of the dimer is a trapezoid formation with extensive negative charge along the core domain’s concave surface. This concave surface structure, also known as a canyon, is well conserved in the Hsp-type subfamily [1,4]. The dimer surface structure of the cap domain results in the formation of a groove that measures ~20 Å in diameter. This bowl structure does not display any charge. Therefore, it is called a hydrophobic bowl in the Hsp-type subfamily (Figure 1C). These surface structural features are considered essential factors for the binding of unstructured proteins [12]. Based on its quaternary structure, SAV0551 is classified as a definite member of the Hsp-type subfamily with chaperone function.

### Comparison of SAV0551 with DJ-1 superfamily proteins

**Figure 2:**
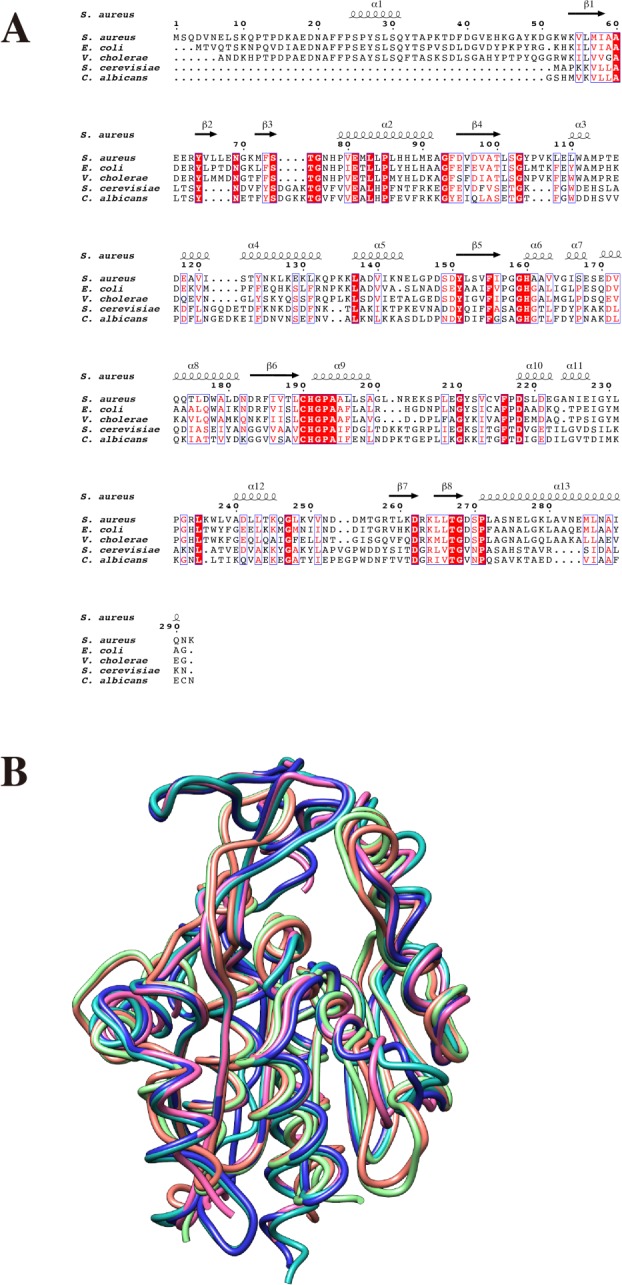
Comparison of SAV0551 with Hsp31 proteins from different origins (**A**) Sequence alignment of SAV0551 with other Hsp31s from different origins. Sequence alignment of SAV0551 with *E. coli* Hsp31 (54% sequence identity), *V. cholerae* Hsp31 (49% sequence identity), *S. cerevisiae* Hsp31 (12% sequence identity), and *C. albicans* Hsp31 (14% sequence identity) proteins. Identical residues are colored white on a red background and similar residues are red on a white background. Secondary-structure elements (springs are α-helices and arrows are β-strands) are represented above the sequences and are numbered. The figure was constructed using ESPript [41,42]. (**B**) The superposition of SAV0551 (sea green) on the *E. coli* Hsp31 (pink, PDB code: 1PV2), *V. cholerae* Hsp31 (blue, PDB code: 4I4N), *S. cerevisiae* Hsp31 (green, PDB code: 1QVV), and *C. albicans* Hsp31 (orange, PDB code: 4LRU) structures. The overall fold of SAV0551 is similar to the other Hsp31 proteins. The figure was constructed using UCSF Chimera [33].

The overall fold of SAV0551 monomer shows structural similarity to other Hsp-type subfamily proteins as predicted from the sequence homology, including *E. coli* Hsp31 (54% sequence identity), *V. cholera* Hsp31 (49% sequence identity), *S. cerevisiae* YDR533c (12% sequence identity), and *C. albicans* Glx3 (14% sequence identity). A structure-based sequence alignment was acquired using the Clustal Omega web server tool [39,40] and viewed using the ESPript web server tool [41,42]. The overall fold of SAV0551 is similar to other Hsp-type subfamily members. The SAV0551 structure was submitted to the DALI server (http://ekhidna.biocenter.helsinki.fi/dali_server/) to identify structural homologs. The DALI algorithm reveals that SAV0551 is structurally similar to Hsp31 proteins with high Z scores. Most of the structural matches were DJ-1 family members, including Hsp31 from *V. cholerae* (Z score =46.5, 0.88 r.m.s.d., 277 equivalent Cα, 49% sequence identity), Hsp31 from *E. coli* (Z score =45.2, 0.73 r.m.s.d., 226 equivalent Cα, 54% sequence identity), YDR533c from *S. cerevisiae* (Z score =24.2, 1.85 r.m.s.d., 196 equivalent Cα, 12% sequence identity), and Glx3 from *C. albicans* (Z score =23.8, 2.12 r.m.s.d., 200 equivalent Cα, 14% sequence identity). The other matches, such as Hsp33 from *S. cerevisiae* (Z score =23.5, 1.97 r.m.s.d., 189 equivalent Cα, 27% sequence identity), DJ-1 from human (Z score =19.6, 2.32 r.m.s.d., 139 equivalent Cα, 27% sequenced identity) and protease *Pf*pI from *P. horikoshii* (Z score =18.9, 1.70 r.m.s.d., 158 equivalent Cα, 23% sequence identity), were also identified. Sequence alignment and superposition of the SAV0551 structure with the Hsp31 proteins from *E. coli* and *V. cholerae*, YDR533c from *S. cerevisiae*, and Glx3 from *C. albicans* are shown in Figures 2A,B, respectively.

**Figure 3:**
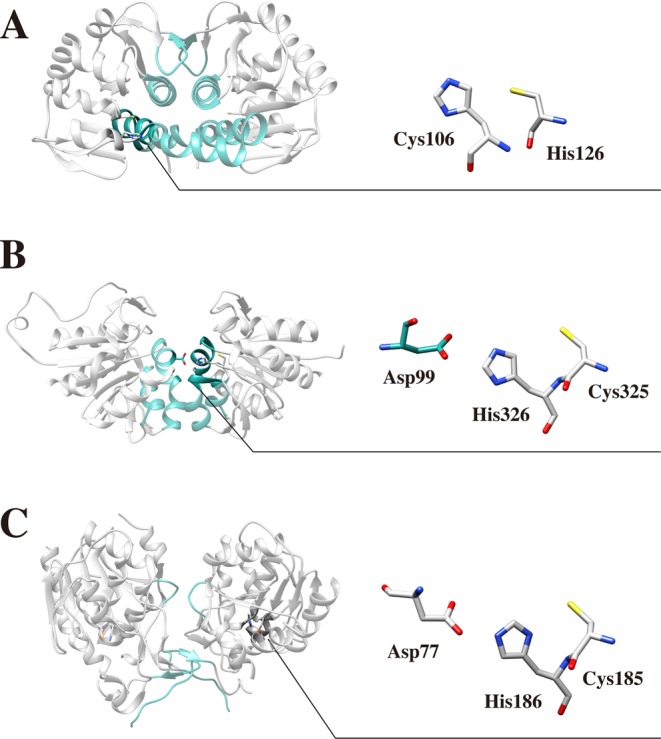
Differences in the dimerization modes and active site configurations amongst the three subfamilies of DJ-1/ThiJ/*Pf*pI superfamily (**A**) In the human DJ-1-type protein DJ-1 (PDB ID: 1P5F), three helices and a loop are involved in dimerization. The cysteine forms a catalytic dyad with a neighboring histidine. (**B**) In the *E. coli* YhbO-type protein YhbO (PDB ID: 1IO4), three helices are responsible for dimerization. The cysteine forms a catalytic triad with the histidine next to the cysteine and an aspartate from the adjacent subunit. (**C**) In the *E. coli* Hsp-type protein Hsp31 (PDB ID: 1PV2), the cap domain is critical for dimerization. The cysteine constitutes a catalytic triad with the histidine next to the cysteine, and an aspartate from the cap domain. The catalytic triad maintains the same handedness, but the backbone orientation of the aspartate differs from YhbO-type subfamily proteins.

Although the tertiary structures of DJ-1 superfamily members are similar, quaternary structures vary according to the DJ-1 subfamilies (DJ-1-, YhbO-, and Hsp-types). DJ-1-type subfamily proteins form dimer using α-helices, β-strands, and loops. YhbO-type subfamily proteins use three α-helices to form a dimer, and Hsp-type subfamily members interact with other subunit by N-terminal β-strands and loops [4]. In addition to the difference in the quaternary structure, another obvious difference is the architecture of the catalytic triad. Although all DJ-1 superfamily proteins have a reactive cysteine at a sharp turn between a β-strand and an α-helix, this cysteine forms different active site constellations depending on the subfamilies. The catalytic triad is not detected in DJ-1-type subfamily proteins. The DJ-1 type DJ-1 forms a catalytic dyad with a nearby histidine [43]. The DJ-1-type protein YajL does not form a catalytic dyad/triad [7]. YhbO-type proteins constitute a catalytic triad with cysteine, the histidine next to the cysteine, and an acidic residue from the other subunit. The Hsp-type proteins form a catalytic triad using the cysteine, the histidine next to the cysteine, and an acidic residue from an intrasubunit from the cap domain [4]. YhbO-type and Hsp-type subfamily proteins have an analogous catalytic triad, though the triads differ in the orientation of the acidic residue (Figure 3). Because the constellation of the three residues in the catalytic triad is identical between YhbO-type and Hsp-type subfamily proteins, the bond angles and distances between the atoms are similar. Although it is known that the catalytic triad may contribute to the protease and glyoxalase III mechanisms in the YhbO-type and Hsp-type subfamily, respectively, the exact function of the catalytic triad in the DJ-1 superfamily has not yet been revealed. In that sense, this structural difference may implicate the function of the catalytic triad and type of potential substrates.

### Chaperone activity of SAV0551 and its mutants

**Figure 4:**
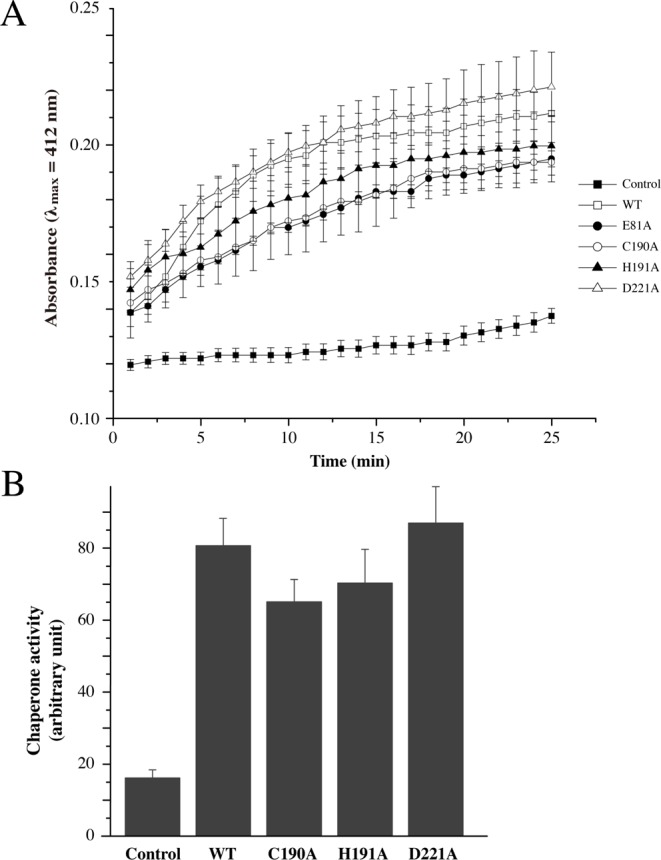
Chaperone activities of wild-type SAV0551 and the catalytic triad mutants SAV0551 exerts a chaperone function. The chaperone activity was assayed by monitoring an increase in the absorbance at 412 nm. (**A**) The control (▪) contained 50 μl of reaction mixture containing 1 mM DTNB, 0.2 mM MnCl_2_, 0.4 mM oxaloacetic acid, and 0.3 mM acetyl-CoA in 100 mM Tris buffer (pH 8.0), with an additional 0.75 μg of denatured citrate synthase. A protein final concentration of 5 μM was used for the wild-type (□), C190A (•), H191A (○), and D221A (▲) protein. (**B**) Calculation of the chaperone activity at the 25-min time point. The data from three scans were averaged.

Hsp-type DJ-1 subfamily proteins are highly conserved in many bacterial species and function as molecular chaperones that facilitate protein folding. Under stress conditions, such as heat shock, pH shift, or oxidation, the increased expression of chaperone proteins protects cells by stabilizing unfolded proteins. Hsp31 proteins are known as holdases, which holding molecular chaperones. The surface structure of Hsp31 is crucial for holding unfolded substrates. Hsp31 from *E. coli* work as a dimer by forming a deep acidic canyon and bowl at its dimeric interfaces [44]. When the surfaces of Hsp31 from *E. coli* were viewed, hydrophobic patches were detected around the canyon for the binding of unstructured proteins. SAV0551 expresses similar surface patterns to Hsp31 from *E. coli*, including a canyon and a bowl. SAV0551 has a deep acidic canyon that winds from the dimeric interface to each side of the subunit, and bowl structure is located on the bottom side (Figure 1C). This characteristic surface structure is well conserved in Hsp-type DJ-1 subfamily members. Other types of DJ-1 subfamily proteins have also shown chaperone activity including the DJ-1-type DJ-1 and the YhbO-type SAV1875 [8,19]. Even though the whole cap domain is absent from the DJ-1-type or YhbO-type subfamily members, the overall surface structure are conserved. Considering that the mechanism of chaperone activity is dependent upon the surface structure, the catalytic triad is not shown to be relevant to its action. To determine the chaperone activity of SAV0551 and any correlation between the catalytic triad and chaperone activity, catalytic triad mutants C190A, H191A, and D221A were designed to identify the structural and functional differences of SAV0551. The observed data showed a chaperone-facilitated renaturation of citrate synthase with the wild-type and SAV0551 mutants (Figure 4). There were no significant differences in the chaperone activity of the wild-type and SAV0551 mutants. From the present study, we identified that SAV0551 is an *S. aureus* chaperone protein and that the presence of cysteine is not a key element for the chaperone function.

### Glyoxalase III activity of SAV0551 and its mutants

Endogenous methylglyoxal is generated as an unavoidable consequence of glycolysis. It is also formed by lipid peroxidation systems, acetone metabolism, and DNA degradation [22,45,46]. Methylglyoxal is a known endogenous and environmental mutagen that can modify both DNA and proteins. To detoxify methylglyoxal, there is a glyoxalase system that converts methylglyoxal to non-toxic D-lactate. The traditional glyoxalase system is accomplished by the sequential action of the two thiol-dependent enzymes glyoxalase I and II in the presence of glutathione (GSH) [47]. Recently, a GSH-independent glyoxalase system that utilizes Hsp31 was identified in *E. coli*. In this system, the *E. coli* Hsp31 directly converts methylglyoxal to D-lactate in a single step, independent of GSH [15].

**Figure 5:**
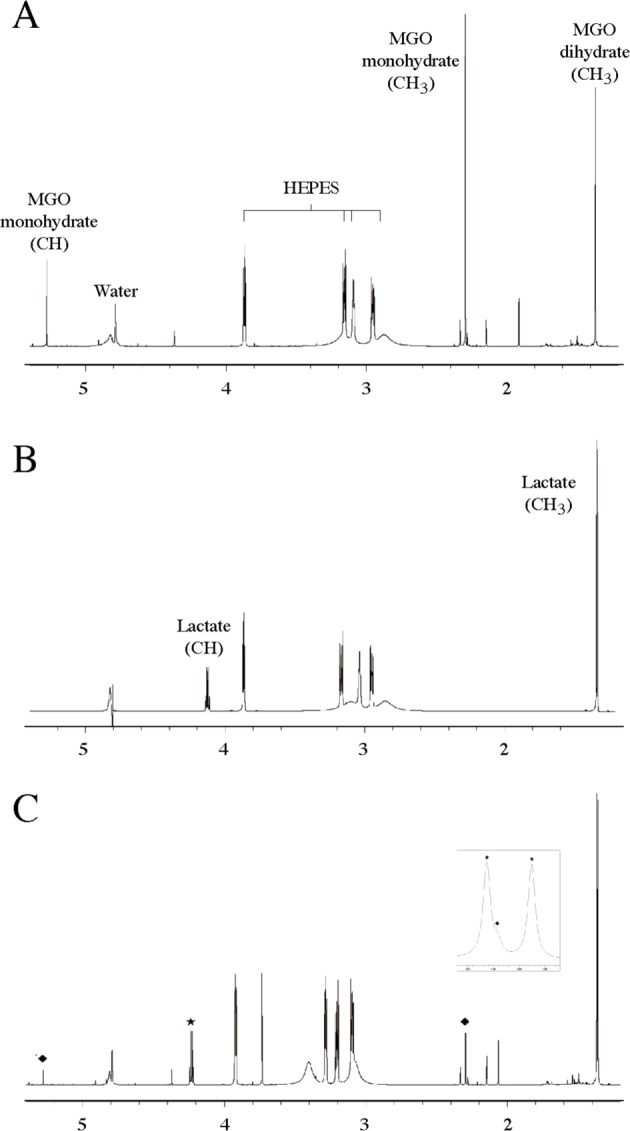
NMR-based assessment of SAV0551 glyoxalase III activity (**A**) 1D ^1^H NMR spectrum for methylglyoxal. (**B**) 1D ^1^H NMR spectrum for D-lactate. (**C**) 1D ^1^H NMR spectrum for D-lactate produced by SAV0551. Newly generated and decreased peaks are represented by a ‘★’ and ‘♦’, respectively. The peak at 3.73 ppm is derived from the Tris buffer in which the protein stock is dissolved. An expanded view of the peaks for MGO dihydrate (CH_3_) and -lactate (CH_3_) are shown in the rectangular box. The reaction was performed at 37°C in 20 mM HEPES (pH 7.5) 50 mM KCl, and 2 mM DTT. The reaction concentrations of SAV0551 and its substrate methylglyoxal were 50 μM and 60 mM, respectively.

To determine the glyoxalase III activity of SAV0551, 1D NMR experiments were used. The enzymatic reaction could be analyzed by identifying newly generated product peaks. Due to the different monomeric and polymeric structures formed by methylglyoxal in H_2_O, all the corresponding proton peaks appeared in the conventional 1D NMR spectrum of methylglyoxal (Figure 5A). The assignment of these peaks was performed on the basis of the previous resonance assignment of methylglyoxal and its derivatives as well as with the ACD/NMR program predictor [48,49]. The spectrum predominantly showed three peaks for monohydrated and dihydrated monomeric forms of methylglyoxal; the methyl protons (CH_3_) of the monohydrate and dihydrate correspond to resonances at 2.292 and 1.363 ppm, respectively, and the alkyl proton (CH) of the monohydrate corresponds to a resonance at 5.275 ppm. The peak for the dihydrate alkyl proton was not observed because its signal at ~4.8 ppm was suppressed by the water signal. Many low-intensity peaks for the polymeric forms mainly appeared in a range from 1.0 to 1.8 ppm. The reference spectrum of D-lactate in the same buffer was acquired to assign its chemical shifts (Figure 5B). The two main proton peaks for D-lactate appeared at ~4.12 ppm (CH) and 1.337 ppm (CH_3_). After the enzymatic reaction of methylglyoxal with SAV0551, the 1D spectrum of this solution was obtained as shown in Figure 5C. We observed new peaks that correspond to the D-lactate produced by SAV0551 as well as a significant reduction in the peak intensities of methylglyoxal monohydrate and dihydrate. This result demonstrated that the two hydrated forms of methylglyoxal can be used as a substrate for SAV0551 to produce D-lactate, which is consistent with our data obtained by absorption spectroscopy. Additionally, the peak for Tris buffer, in which the proteins were dissolved, appeared at 3.73 ppm; the peaks for HEPES buffer were slightly changed, possibly because the production of D-lactate caused a decrease in the pH. An unassigned peak at 1.91 ppm, which might come from a methylglyoxal derivative, increased to 2.06 ppm.

**Figure 6:**
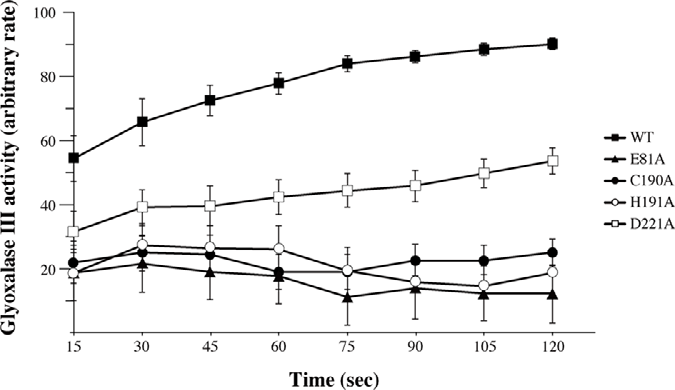
Glyoxalase III activity of the wild-type and SAV0551 mutants SAV0551 degrades methylglyoxal. Thus, 6 mM methylglyoxal was used initially, and the remaining amount of methylglyoxal was calculated after incubation with the wild-type and E81A, C190A, H191A, and D221A mutant proteins over time.

In addition to the 1D NMR spectroscopy, the glyoxalase III activities of SAV0551 and its mutants were assessed by absorption spectroscopy. The amounts of methylglyoxal were detected at an absorbance of 550 nm after the reaction with DNPH to generate the purple chromophore methylglyoxal-bis-2,4-dinitrophenylhydrazone after alkali treatment [16]. The wild-type and SAV0551 mutants were employed as the enzymes. The levels of methylglyoxal were substantially reduced by the time when it was mixed with wild-type SAV0551. However, the glyoxalase III activities of the SAV0551 mutants were diminished (Figure 6). The E81A, C190A, and H191A mutants showed little or no activities, while the D221A mutant displayed marginal activity. From our results, SAV0551 is determined to be a glyoxalase III and the key residues involved in its function are Glu^81^ and catalytic triad residues Cys^190^, His^191^, and Asp^221^.

### Components required for glyoxalase III function

The mechanism of the traditional glyoxalase system, which includes glyoxalase I and II, is well known, and cofactors such as GSH and metal ions are necessary for the reaction [50–52]. However, even though the substrate and product are the same, glyoxalase III does not require any cofactors. According to the known glyoxalase III mechanism via *Arabidopsis thaliana* DJ-1, the SAV0551 glyoxalase III mechanism is expected to be performed by the residues in the active site, including the catalytic triad (Cys^190^, His^191^, and Asp^221^), an absolutely conserved glutamate amongst the DJ-1 superfamily (Glu^81^), and a base residue histidine residue that is positioned differently compared with the structure of *A. thaliana* DJ-1 [52]. The characteristic aspect of the glyoxalase III mechanism is that the enzyme itself has all the required components within the active site; therefore, it functions without any cofactors.

To produce D-lactate from methylglyoxal, a ketone carbonyl group (the C2 carbonyl group) is reduced to a hydroxyl group, while the aldehyde carbonyl group is oxidized to an alcohol. When the glyoxalase III mechanism is compared with the traditional glyoxalase system (glyoxalases I and II), the initial nucleophilic reaction is completed by the catalytic cysteine instead of GSH, which is an essential cofactor in the traditional glyoxalase system. Once the hemithioacetal forms, glyoxalase I moves the proton from C1 to C2 and generates S-D-lactoylgluthathione. The Zn^2+^ ions stabilize the enediol intermediate to lower the free energy of proton transfer, which finally leads to the production of S-D-lactoylglutathione [53,54]. In the *A. thaliana* DJ-1 glyoxalase III mechanism, the backbone glycine stabilizes O1_C1_ and an absolutely conserved glutamate stabilizes O2_C2_ so that the proton can transfer from C1 to C2 via the glutamate residue, similar to the glyoxalase I mechanism. The histidine residues in the glyoxalase III active site substitute for the action of the Zn^2+^ ions. The final step is hydrolysis, which is the function of glyoxalase II, and metal ions are also required for the introduction of the water ions. Once again, the histidine residue holds the water molecule to aid in the hydrolysis.

**Figure 7:**
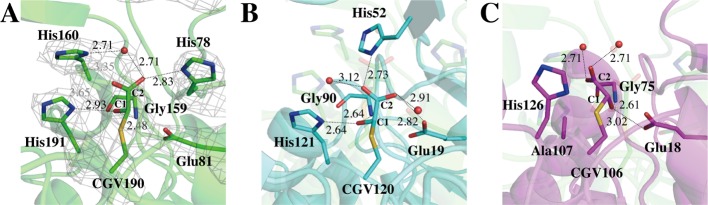
Catalytic triad and surrounding residues around the Cys active site The glyoxylate-bound co-crystal structures of SAV0551, *A. thaliana* DJ-1, and human DJ-1. Distances are given in Angstroms. Hydrogen bonds are shown in dark dotted lines. (**A**) Chain A in the glyoxylate-bound structure of SAV0551. Unlike the O1_C1_ and O2_C2_ oxygen atoms, the glyoxylate C2 oxygen atom (O3_C2_) does not form stable hydrogen bonds. The 2*Fo-Fc* electron density map contoured at a level of 1.0σ is shown in gray. (**B**) The *A. thaliana* DJ-1 co-crystal structure with glyoxylate (cyan, PDB code: 4OGG) and the corresponding region in SAV0551 (light green). All three glyoxylate oxygen atoms form stable hydrogen bonds. (**C**) The glyoxylate co-ordination in human DJ-1 (magenta, PDB code: 4OGF) and the corresponding region in SAV0551 (light green). The co-crystal structure with glyoxylate is stabilized by hydrogen bonds through the introduction of water molecules.

In light of the structural comparison between *A. thaliana* DJ-1 and SAV0551, the active site residues are well conserved, except for the base histidine. Although the *A. thaliana* DJ-1 histidine residue (His^52^) is proven to be crucial for the glyoxalase III function, the geometry of the histidine residues is different in SAV0551 crystal structure compared with *A. thaliana* DJ-1. Two histidine residues (His^78^ and His^160^) are positioned in the active site of SAV0551 (Figures 7A,B).

### Structural study of the SAV0551 active site as a glyoxalase III

To secure the SAV0551 glyoxalase III reaction intermediate and to determine the difference in the histidine location between *A. thaliana* DJ-1 and SAV0551, SAV0551 was crystallized with the DJ-1 glyoxalase III inhibitor glyoxylate. The glyoxylate is stably co-ordinated in the active site of *A. thaliana* DJ-1 and *Homo sapiens* DJ-1. In the *A. thaliana* DJ-1 co-crystal structure, the glyoxylate C1 carbon–oxygen atom (O1_c1_) forms stable hydrogen bonds with His^121^ (2.64 Å) and Gly^90^ (2.64 Å). The other oxygen (O2_c2_) atom at the C2 carbon is stabilized by Glu^19^ (2.82 Å) and a water molecule (2.91 Å). The third oxygen atom (O2_c3_), which is a methyl group in the substrate methylglyoxal structure, also shows hydrogen bonds with His^52^ (2.73 Å) and a water molecule (3.12 Å) (Figure 7B). In the human DJ-1 co-crystal structure, the oxygen atom at C1 (O1_C1_) forms stable hydrogen bonds with Glu^18^ (3.02 Å) and Gly^75^ (2.61 Å). The two oxygen atoms at C2 show hydrogen bonds with water molecules (2.71 and 2.71 Å, respectively) (Figure 7C) [52]. However, the glyoxylate in the SAV0551 crystal structure is not perfectly co-ordinated, potentially because of the relatively low resolution. Still, the SAV0551 co-crystal structure shows the clear density of the glyoxylate C1 and the interaction with Glu^81^ and Gly^159^. With this information, the rigid glyoxylate structure is well positioned with respect to the active site. Amongst the three glyoxylate oxygen atoms, O3_C2_ does not form hydrogen bonds, while O1_C1_ and O2_C2_ show stable hydrogen bonds. O1_C1_ forms a hydrogen bond with His^191^ (2.93 Å) and Gly^159^ (2.48 Å). The C2 carbon–oxygen atom (O2_C2_) forms a hydrogen bond with His^78^ (2.83 Å) and a water molecule (2.71 Å). The third oxygen atom (O3_C2_) is 3.35 Å from a water molecule and 3.65 Å from His^191^, which demonstrate weak electrostatic bonds. Because of the different constellation of the residues in the SAV0551 and *A. thaliana* DJ-1 active sites, the glyoxylate co-ordination in SAV0551 can be limited because the O3_C2_ is not stabilized by residues or water molecules. The glyoxylate-bound crystal structure in SAV0551 is similar to the glyoxalase III reaction intermediate (hemithioacetal) because the glyxalase III substrate methylglyoxal has a single oxygen atom on C2. The O3_C2_ is a methyl group in methylglyoxal, and the methyl group does not form any hydrogen bonds. From the glyoxylate-bound structure, we determined that His^78^ provides a hydrogen atom to the O2_C2_ and that a water molecule attacks the C1 atom to hydrolyze the D-lactoylcysteine structure to produce D-lactate. Therefore, we can conclude that SAV0551 is a glyoxalase III that possesses all the required components in its active site, including His^78^, which acts as a base in the glyoxalase III mechanism. Additionally, in the SAV0551 co-crystal structure, excess amounts of glyoxylate are also located in the aspartate-rich regions within the adjacent cleft of two irrelevant chains, which are not dimers.

## Discussion

The structural studies herein confirmed SAV0551 from *S. aureus* Mu50 as a member of the DJ-1 superfamily and that it has all the pertinent traits of this group, including the sandwich fold and nucleophilic arm. It specifically belongs to the Hsp-type subfamily because of the presence of cap domain and aspects of its dimeric interface. Simultaneously, Hsp-type DJ-1 subfamily proteins are categorized as Class 20 (Hsp31) chaperones by the recent classification of small HSPs (sHSP) [55]. Hsp31 from *E. coli* functions as a holding chaperone and displays chaperone activity by holding unfolded protein with its canyon, bowl, and hydrophobic surface structure [12]. SAV0551 has a similar canyon, bowl, and hydrophobic surface structure as *E. coli* Hsp31, suggesting that SAV0551 may work as a chaperone. We discovered that the wild-type and SAV0551 mutants assisted in the folding of citrate synthase, indicating the importance of overall its structure rather than specific residues.

Accumulation of methylglyoxal causes endogenous damage by reacting with proteins and nucleic acids. The DJ-1 superfamily, especially Hsp-type DJ-1 subfamily proteins, is well known for its role in the cellular defense system toward methylglyoxal toxicity. SAV0551 is also predicted to perform similar biological functions as members of the Hsp-type subfamily. To determine the glyoxalase activity, 1D NMR and absorption spectroscopy experiments were performed. The co-crystal structure with the known inhibitor glyoxylate was also determined to elucidate the structure of the intermediate. The results demonstrate that SAV0551 is a glyoxalase III that produces non-toxic D-lactate from toxic methylglyoxal independent of any cofactors. We discovered that residues in the catalytic triad (Cys^190^, H191, and D221), an absolutely conserved Glu^81^, and His^78^ are responsible for the glyoxalase III function. The catalytic cysteine (Cys^190^) attacks the methylglyoxal aldehyde group (C1) and forms a hemithioacetal intermediate. Then, Glu^81^ moves a proton from C1 to C2 via stabilization of the enediol through the neighboring residues His^78^, Gly^159^, and His^191^ and a water molecule. After the formation of D-lactoylcysteine, a water molecule attacks C1 and hydrolyzes it to produce ^D^-lactate. In the present study, we have shown the significance of the catalytic triad residues (Cys^190^, H191, and D221) and the absolutely conserved glutamate residue (Glu^81^) in the DJ-1 superfamily and revealed the important base residue His^78^ via a co-crystal structure.

In summary, the crystal structure of the Hsp-type protein SAV0551 and a co-crystal structure with glyoxylate were determined. SAV0551 has the distinctive characteristic structure of a holding chaperone, including the oligomeric state, surface canyon, and bowl structure and is similar to *E. coli* Hsp31. SAV0551 shows chaperone activity regardless of active site mutations, whereas the catalytic cysteine and active site residues are crucial for its glyoxalase III function. Because DJ-1 superfamily proteins have multiple stress defense functions including chaperone and glyoxalase III, our study will promote a better understanding of how bacteria can defend against stress conditions. By focussing on DJ-1 superfamily proteins such as SAV0551, we can gain insights into the essential cellular stress control systems against stress condition. Future research on DJ-1 family proteins and *in vivo* studies are required to fully understand the fundamentals of stress response functions.

## Abbreviations

DTT: dithiothreitol
DNPH: 2,4-dinitrophenylhydrazine
DTNB: 5,5′-dithiobis-(2-nitrobenzoic acid)
GSH: glutathione
HSP: heat shock protein

## References

[1] Wei Y. (2007) Identification of functional subclasses in the DJ-1 superfamily proteins. PLoS Comput. Biol. 3, e10 17257049 10.1371/journal.pcbi.0030010 PMC1782040

[2] Tang B. (2006) Association of PINK1 and DJ-1 confers digenic inheritance of early-onset Parkinson’s disease. Hum. Mol. Genet. 15, 1816–1825 16632486 10.1093/hmg/ddl104

[3] Wang Z.Q., Zhou H.Y. and Chen S.D. (2006) The role of DJ-1 in the pathogenesis of Parkinson’s disease. Neurosci. Bull. 22, 232–234 17704838

[4] Jung H.J. (2012) Dissection of the dimerization modes in the DJ-1 superfamily. Mol. Cells 33, 163–171 22228183 10.1007/s10059-012-2220-6 PMC3887719

[5] Ollis D.L. (1992) The alpha/beta hydrolase fold. Protein Eng. 5, 197–211 1409539 10.1093/protein/5.3.197

[6] Honbou K. (2003) The crystal structure of DJ-1, a protein related to male fertility and Parkinson’s disease. J. Biol. Chem. 278, 31380–31384 12796482 10.1074/jbc.M305878200

[7] Wilson M.A., Ringe D. and Petsko G.A. (2005) The atomic resolution crystal structure of the YajL (ThiJ) protein from *Escherichia coli*: a close prokaryotic homologue of the Parkinsonism-associated protein DJ-1. J. Mol. Biol. 353, 678–691 16181642 10.1016/j.jmb.2005.08.033

[8] Shendelman S. (2004) DJ-1 is a redox-dependent molecular chaperone that inhibits alpha-synuclein aggregate formation. PLoS Biol. 2, e362 15502874 10.1371/journal.pbio.0020362 PMC521177

[9] Lee J.Y. (2012) Human DJ-1 and its homologs are novel glyoxalases. Hum. Mol. Genet. 21, 3215–3225 22523093 10.1093/hmg/dds155

[10] Zhang Q. (2012) DJ-1 promotes the proteasomal degradation of Fis1: implications of DJ-1 in neuronal protection. Biochem. J. 447, 261–269 22871147 10.1042/BJ20120598

[11] Zhan D. (2014) Characterization of the PH1704 protease from *Pyrococcus horikoshii* OT3 and the critical functions of Tyr120. PLoS ONE 9, e103902 25192005 10.1371/journal.pone.0103902 PMC4156298

[12] Quigley P.M. (2003) The 1.6-Å crystal structure of the class of chaperones represented by *Escherichia coli* Hsp31 reveals a putative catalytic triad. Proc. Natl. Acad. Sci. U.S.A. 100, 3137–3142 12621151 10.1073/pnas.0530312100 PMC152259

[13] Mujacic M., Bader M.W. and Baneyx F. (2004) *Escherichia coli* Hsp31 functions as a holding chaperone that cooperates with the DnaK-DnaJ-GrpE system in the management of protein misfolding under severe stress conditions. Mol. Microbiol. 51, 849–859 14731284 10.1046/j.1365-2958.2003.03871.x

[14] Das S. (2017) Structural and biochemical studies on *Vibrio cholerae* Hsp31 reveals a novel dimeric form and Glutathione-independent Glyoxalase activity. PLoS ONE 12, e0172629 28235098 10.1371/journal.pone.0172629 PMC5325305

[15] Subedi K.P. (2011) Hsp31 of *Escherichia coli* K-12 is glyoxalase III. Mol. Microbiol. 81, 926–936 21696459 10.1111/j.1365-2958.2011.07736.x

[16] Hasim S. (2014) A glutathione-independent glyoxalase of the DJ-1 superfamily plays an important role in managing metabolically generated methylglyoxal in *Candida albicans*. J. Biol. Chem. 289, 1662–1674 24302734 10.1074/jbc.M113.505784 PMC3894345

[17] Zhao Q. (2014) Identification of glutathione (GSH)-independent glyoxalase III from *Schizosaccharomyces pombe*. BMC Evol. Biol. 14, 86 24758716 10.1186/1471-2148-14-86 PMC4021431

[18] Bankapalli K. (2015) Robust glyoxalase activity of Hsp31, a ThiJ/DJ-1/*Pf*pI family member protein, is critical for oxidative stress resistance in *Saccharomyces cerevisiae*. J. Biol. Chem. 290, 26491–26507 26370081 10.1074/jbc.M115.673624 PMC4646309

[19] Kim H.J., Kwon A.R. and Lee B.J. (2016) Structural and functional insight into the different oxidation states of SAV1875 from *Staphylococcus aureus*. Biochem. J. 473, 55–66 26487697 10.1042/BJ20150256

[20] Abdallah J. (2016) The DJ-1 superfamily members YhbO and YajL from *Escherichia coli* repair proteins from glycation by methylglyoxal and glyoxal. Biochem. Biophys. Res. Commun. 470, 282–286 26774339 10.1016/j.bbrc.2016.01.068

[21] Papp E. (2003) Molecular chaperones, stress proteins and redox homeostasis. Biofactors 17, 249–257 12897446 10.1002/biof.5520170124

[22] Allaman I., Belanger M. and Magistretti P.J. (2015) Methylglyoxal, the dark side of glycolysis. Front. Neurosci. 9, 23 25709564 10.3389/fnins.2015.00023 PMC4321437

[23] Archer G.L. (1998) *Staphylococcus aureus*: a well-armed pathogen. Clin. Infect. Dis. 26, 1179–1181 9597249 10.1086/520289

[24] Woods C. and Colice G. (2014) Methicillin-resistant *Staphylococcus aureus* pneumonia in adults. Expert Rev. Respir. Med. 8, 641–651 25030040 10.1586/17476348.2014.940323

[25] Di Nottia M. (2016) DJ-1 modulates mitochondrial response to oxidative stress: clues from a novel diagnosis of PARK7. Clin. Genet. 12841 10.1111/cge.12841 27460976

[26] Otwinowski Z. and Minor W. (1997) Processing of X-ray diffraction data collected in oscillation mode. Methods Enzymol. 276, 307–326 10.1016/S0076-6879(97)76066-X 27754618

[27] Winn M.D. (2011) Overview of the CCP4 suite and current developments. Acta Crystallogr. D Biol. Crystallogr. 67, 235–242 21460441 10.1107/S0907444910045749 PMC3069738

[28] Vagin A. and Teplyakov A. (2010) Molecular replacement with MOLREP. Acta Crystallogr. D Biol. Crystallogr. 66, 22–25 20057045 10.1107/S0907444909042589

[29] Emsley P. and Cowtan K. (2004) Coot: model-building tools for molecular graphics. Acta Crystallogr. D Biol. Crystallogr. 60, 2126–2132 15572765 10.1107/S0907444904019158

[30] Adams P.D. (2010) PHENIX: a comprehensive Python-based system for macromolecular structure solution. Acta Crystallogr. D Biol. Crystallogr. 66, 213–221 20124702 10.1107/S0907444909052925 PMC2815670

[31] Murshudov G.N. (2011) REFMAC5 for the refinement of macromolecular crystal structures. Acta Crystallogr. D Biol. Crystallogr. 67, 355–367 21460454 10.1107/S0907444911001314 PMC3069751

[32] Brunger A.T. (1992) Free R value: a novel statistical quantity for assessing the accuracy of crystal structures. Nature 355, 472–475 18481394 10.1038/355472a0

[33] Pettersen E.F. (2004) UCSF Chimera-a visualization system for exploratory research and analysis. J. Comput. Chem. 25, 1605–1612 15264254 10.1002/jcc.20084

[34] Krissinel E. and Henrick K. (2007) Inference of macromolecular assemblies from crystalline state. J. Mol. Biol. 372, 774–797 17681537 10.1016/j.jmb.2007.05.022

[35] Zhi W. (1992) Renaturation of citrate synthase: influence of denaturant and folding assstants. Protein Sci. 1, 522–529 1363914 10.1002/pro.5560010407 PMC2142213

[36] Lee G.J. (1995) Assaying proteins for molecular chaperone activity. Methods Cell. Biol. 50, 325–334 8531805 10.1016/s0091-679x(08)61040-7

[37] Morgunov I. and Srere P.A. (1998) Interaction between citrate synthase and malate dehydrogenase. Substrate channeling of oxaloacetate. J. Biol. Chem. 273, 29540–29544 9792662 10.1074/jbc.273.45.29540

[38] Gilbert R.P. and Brandt R.B. (1975) Spectrophotometric determination of methyl glyoxal with 2,4-dinitrophenylhydrazine. Anal. Chem. 47, 2418–2422 1190480 10.1021/ac60364a003

[39] Sievers F. (2011) Fast, scalable generation of high-quality protein multiple sequence alignments using Clustal Omega. Mol. Syst. Biol. 7, 539 21988835 10.1038/msb.2011.75 PMC3261699

[40] McWilliam H. (2013) Analysis Tool Web Services from the EMBL-EBI. Nucleic Acids Res. 41, W597–W600 23671338 10.1093/nar/gkt376 PMC3692137

[41] Gouet P. (1999) ESPript: analysis of multiple sequence alignments in PostScript. Bioinformatics 15, 305–308 10320398 10.1093/bioinformatics/15.4.305

[42] Robert X. and Gouet P. (2014) Deciphering key features in protein structures with the new ENDscript server. Nucleic Acids Res. 42, W320–W324 24753421 10.1093/nar/gku316 PMC4086106

[43] Chen J., Li L. and Chin L.S. (2010) Parkinson disease protein DJ-1 converts from a zymogen to a protease by carboxyl-terminal cleavage. Hum. Mol. Genet. 19, 2395–2408 20304780 10.1093/hmg/ddq113 PMC2876885

[44] Sastry M.S. (2004) The linker-loop region of *Escherichia coli* chaperone Hsp31 functions as a gate that modulates high-affinity substrate binding at elevated temperatures. Proc. Natl. Acad. Sci. U.S.A. 101, 8587–8592 15173574 10.1073/pnas.0403033101 PMC423238

[45] Oya T. (1999) Methylglyoxal modification of protein. Chemical and immunochemical characterization of methylglyoxal-arginine adducts. J. Biol. Chem. 274, 18492–18502 10373458 10.1074/jbc.274.26.18492

[46] Kang J.H. (2003) Oxidative damage of DNA induced by methylglyoxal *in vitro*. Toxicol. Lett. 145, 181–187 14581171 10.1016/s0378-4274(03)00305-9

[47] Martins A.M. (2001) In situ kinetic analysis of glyoxalase I and glyoxalase II in *Saccharomyces cerevisiae*. Eur. J. Biochem. 268, 3930–3936 11453985 10.1046/j.1432-1327.2001.02304.x

[48] Nemet I., Vikic-Topic D. and Varga-Defterdarovic L. (2004) Spectroscopic studies of methylglyoxal in water and dimethylsulfoxide. Bioorg. Chem. 32, 560–570 15530996 10.1016/j.bioorg.2004.05.008

[49] Zhang J. (2010) A novel capillary electrophoretic method for determining methylglyoxal and glyoxal in urine and water samples. J. Chromatogr. A 1217, 5124–5129 20580005 10.1016/j.chroma.2010.05.040

[50] Vander Jagt D.L. (1993) Glyoxalase II: molecular characteristics, kinetics and mechanism. Biochem. Soc. Trans. 21, 522–527 8359524 10.1042/bst0210522

[51] Creighton D.J. and Hamilton D.S. (2001) Brief history of glyoxalase I and what we have learned about metal ion-dependent, enzyme-catalyzed isomerizations. Arch. Biochem. Biophys. 387, 1–10 11368170 10.1006/abbi.2000.2253

[52] Choi D. (2014) Stereospecific mechanism of DJ-1 glyoxalases inferred from their hemithioacetal-containing crystal structures. FEBS J. 281, 5447–5462 25283443 10.1111/febs.13085

[53] O’Young J., Sukdeo N. and Honek J.F. (2007) *Escherichia coli* glyoxalase II is a binuclear zinc-dependent metalloenzyme. Arch. Biochem. Biophys. 459, 20–26 17196158 10.1016/j.abb.2006.11.024

[54] Suttisansanee U. (2015) Modulating glyoxalase I metal selectivity by deletional mutagenesis: underlying structural factors contributing to nickel activation profiles. Metallomics 7, 605–612 25557363 10.1039/c4mt00299g

[55] Jaspard E. and Hunault G. (2016) sHSPdb: a database for the analysis of small Heat Shock Proteins. BMC Plant Biol. 16, 135 27297221 10.1186/s12870-016-0820-6 PMC4906601

